# Efficacy of Hyaluronic Acid in Relieving Post-implantation Pain: A Split-Mouth Randomized Controlled Trial

**DOI:** 10.7759/cureus.36575

**Published:** 2023-03-23

**Authors:** Waseem H Alkhateeb, Ammar Mahmoud Mashlah, Mohammad Y Hajeer, Abeer Ahmad Aljoujou

**Affiliations:** 1 Department of Oral Medicine, University of Damascus Faculty of Dentistry, Damascus, SYR; 2 Department of Orthodontics, University of Damascus Faculty of Dentistry, Damascus, SYR

**Keywords:** patient-reported outcome measure, implantation, implants, pain perception, visual analog scale (vas), hyaluronic acid

## Abstract

Background

Many patients suffer from some degree of pain following the surgical procedures of dental implantation. The fear of pain may be one reason for postponing such prosthodontic treatments. Many procedures have been suggested to control post-implantation pain. This trial evaluated the effectiveness of using hyaluronic acid (HA) during dental implantation on patients’ perceived pain during the postsurgical soft-tissue healing period.

Methodology

A split-mouth randomized controlled trial (RCT) was conducted. The trial sample consisted of 22 dental implants in 11 patients (five males and six females). Patients were selected from those attending the Department of Oral Medicine at the Faculty of Dentistry, University of Damascus between February 2021 and May 2022. The implants were performed in similar bone quality and density for each patient as the implants were inserted in the same jaw on both sides to ensure the same physiological conditions. The study sample was divided into two groups. The first group (the experimental group) consisted of 11 implants in which the implant site was drilled, following which HA was placed inside the implant site and on the surrounding bone before the flap was returned and sutured. The second group (the control group) comprised 11 implants following the conventional procedure without applying any material to the implant socket. The main outcome measure was pain perception which was assessed using the visual analog scale (VAS). Patients were asked to record their perceived pain on the first, third, and tenth days. Two-sample t-tests were used to detect significant differences.

Results

There were statistically significant differences in the mean pain intensity between the experimental and control groups on the first, third, and tenth days (p < 0.05). The mean values of perceived pain in the control group were 5.68, 1.72, and 0.56 on the first, third, and tenth days, respectively. In comparison, the mean values of perceived pain in the experimental group were 4.52, 1.14, and 0.18 on the first, third, and tenth days, respectively. The maximum perceived pain in the control group was 7.5 on the first day following implantation, whereas the maximum value recorded in the experimental group was 6.5. At the third assessment time (i.e., 10 days following the surgical intervention), the mean values were in the very mild category of pain intensity.

Conclusions

This study showed that applying HA in the implant cavity and on the surrounding bone effectively reduced pain after dental implant surgery in comparison with the control group. Patients had lower mean pain scores at one, three, and ten days following surgery compared to the conventional method. HA is suggested to be an adjunctive method to control postsurgical pain after dental implantation.

## Introduction

Dental implantation is a surgical procedure in which a piece fixed to the jaw bones is placed to support a crown, bridge, or movable device [1]. The success of any implant depends on a series of factors related to the patient and the procedure itself, such as general health, bio-acceptance of the implant material, implant surface treatment, surgical procedure, local bone quality, and quantity [2]. Several studies have been conducted on the use of auxiliary materials that can be applied in the context of dental implants to ensure higher success rates and benefit from the additional properties of these materials [3]. For example, hyaluronic acid (HA), which effectively transfers some elements that direct bone growth and tissue regeneration and accelerate bone restoration, has been reported [4].

The body naturally produces HA which forms polysaccharide chains found in the extracellular space of connective tissue, synovial fluid, and other tissues [4]. It has many structural and physiological functions, including intra- and extracellular interactions, interactions with growth factors, and osmotic pressure regulation, as these functions help maintain tissue structure and integrity [4]. In general, all cells of the body, especially connective tissues, can produce HA, as it is synthesized in the cell membrane and then excreted into the extracellular matrix [5].

Studies have shown that a high concentration of low or moderate-molecular-weight HA molecules has the strongest effect in controlling germs compared to other elements [6]. It has an anti-inflammatory effect in the initial stages of inflammation and promotes the infiltration of inflammatory cells and the extracellular space into the wound site [7]. In addition to activating cell migration, reproduction, and differentiation, HA helps differentiate the cells responsible for rebuilding the damaged tissue and plays an important role in forming blood vessels [8]. It accelerates bone repair through chemotaxis, proliferation, and successive differentiation of mesenchymal cells, as well as supports the growth of fibroblasts, chondrocytes, and mesenchymal stem cells [7]. HA has been used as a direct injection into the articular space of the temporomandibular joint [9]. It has also been used to treat lichen planus [10]. HA has been used as an adjunctive therapy during the surgical correction of Class I Miller gingival recession [11]. Moreover, it has been used to relieve complications such as impacted mandibular third molar surgery pain, trismus, edema, and dry sockets [12].

When reviewing the literature, some studies have evaluated the use of HA in relieving pain and inducing new bone formation. A study by Yilmaz et al. aimed to evaluate the effectiveness of applying HA topically to relieve the complications of extraction of the impacted lower third molars [13]. Because the pain decreased in the test group, HA was suggested as an alternative to analgesics after extraction of the impacted mandibular third molars [13]. In another study by Alcantara et al., in which they studied the effect of 1% HA on bone formation in the dental alveolar socket after extraction, it was found that there were significant differences in bone formation in favor of the group where HA gel was applied [14]. In a study by Mohammad and Al-Ghaban to study the effect of HA gel on osseointegration around titanium implants in rabbits, the authors concluded that HA was a bone-conducting substance that promoted and accelerated fusion around titanium implants by stimulating osteoblasts and early localization of bone tissue [15].

Few previous studies have used HA in the implant socket, and most used HA topically after the placement of dental implants in the form of ointments and sprays [16,17]. In these studies, the application was made following suturing. However, in this study, the intention was to inject HA in the implant socket and on the alveolar bone before implant placement and suturing to preserve HA for a long time. This study aimed to evaluate the effectiveness of using HA in dental implants in terms of pain relief that may accompany dental implant surgery using the visual analog scale (VAS) on the first, third, and tenth days following the surgical intervention.

## Materials and methods

Study design and settings

The design of this study was a split-mouth randomized controlled trial. This trial was conducted between February 2021 and May 2022 at the Department of Oral Medicine, Faculty of Dentistry, University of Damascus. The Faculty of Dentistry at the University of Damascus Local Research Ethics Committee approved this study (UDDS-3255-13112021/SRC-1198). This trial was retrospectively registered at Clinical Trials.gov (NCT05776290).

Sample size

The sample size was calculated using the G*power 3.1.7 program (the Heinrich-Heine University in Düsseldorf, Germany) with a significance level of 0.05 and statistical power of 80%. After applying the calculation assumptions, it was found that 22 implants were required (i.e., 11 patients having two opposite implants in their mouths).

Patient recruitment and follow-up

After examining 47 patients attending the Department of Oral Medicine, Faculty of Dentistry University Damascus, 15 patients (aged 27 to 60) met the requirements for inclusion. Patients were informed about the research using standardized and well-detailed information sheets. Upon accepting to participate in the trial, informed consent forms were obtained from patients. Of the 19 patients who consented to participate in the trial, 11 (five men and six women) were chosen randomly. The inclusion criteria were (1) bilateral tooth loss with sufficient amount of bone volume; (2) no general problems; (3) good oral health; and (4) age between 20 and 60 years. The exclusion criteria were (1) the use of immunosuppressive drugs and corticosteroids for long periods; (2) the existence of serious systemic disorders; (3) contraindications for local anesthesia or oral surgery; (4) pregnant women and nursing mothers; (5) patients receiving chemotherapy or radiation; and (6) alcoholics and heavy smokers.

Experimental and control groups

**Figure 1:**
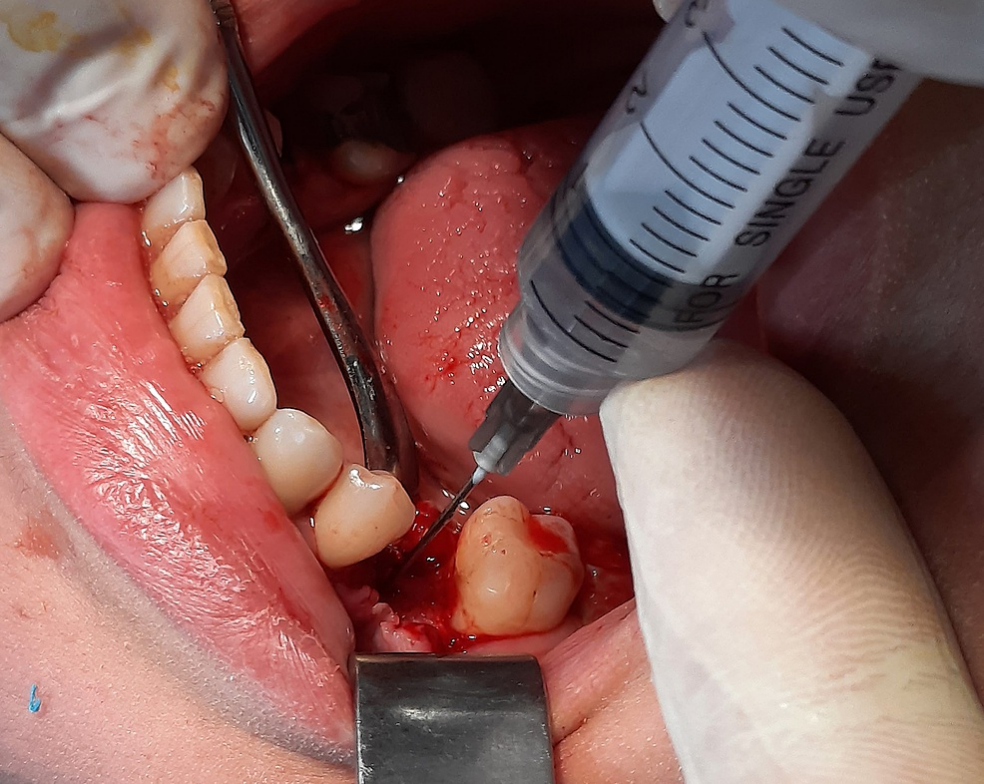
Injection of hyaluronic acid into the alveolar socket before implantation on the experimental side.

**Figure 2:**
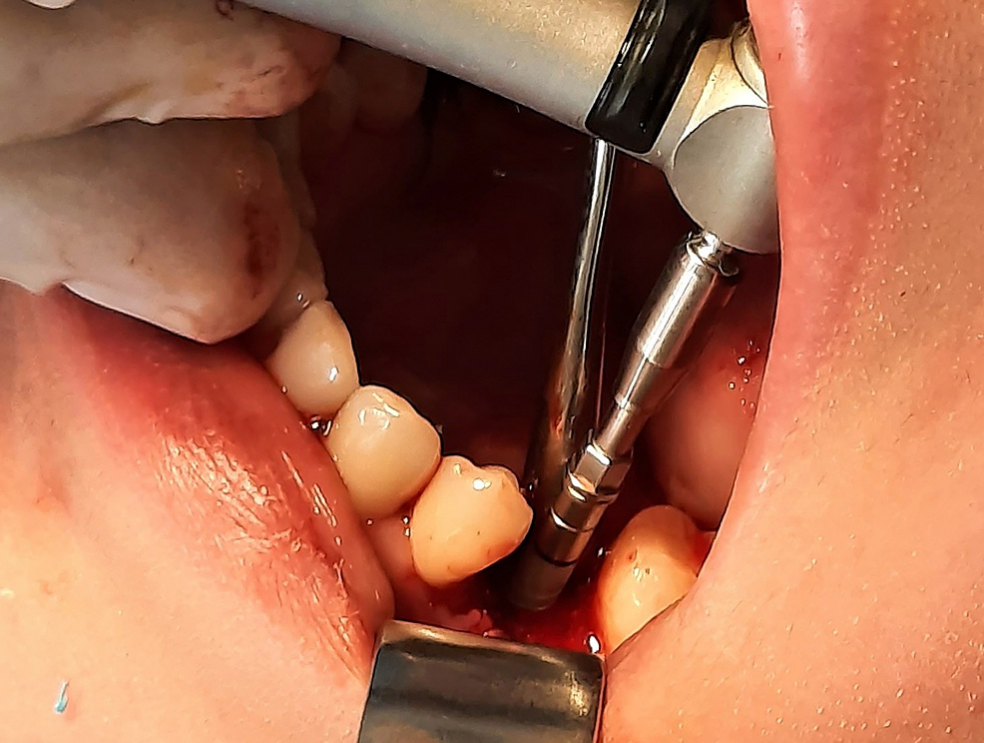
Inserting the implant into the alveolar socket and positioning it at the desired level.

**Figure 3:**
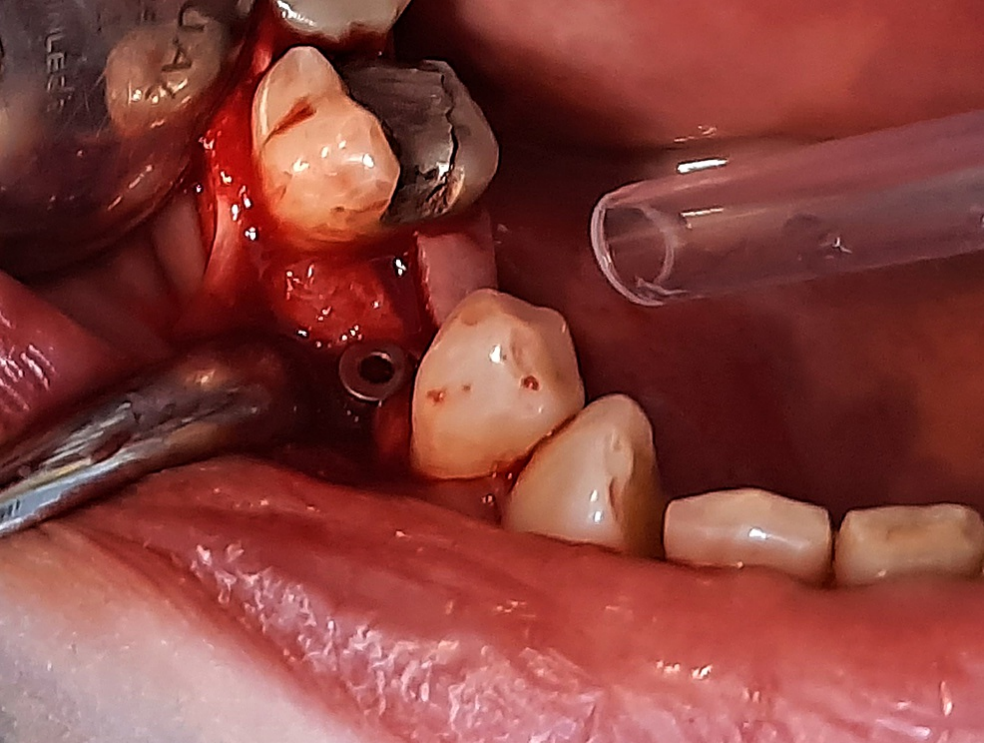
The opposite side being treated conventionally in the control group.

**Figure 4:**
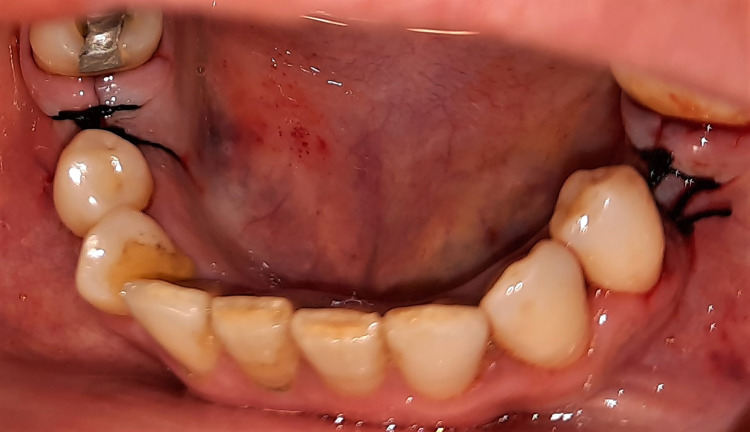
Suturing performed at the end of the implantation procedure in both groups (i.e., on both sides).

A total of 11 patients were included in this trial, with 22 implants implanted, two implants in each patient’s mouth through a split-mouth design. The experimental group consisted of 11 implant cases. The skin around the mouth was initially cleaned using a polyvidone iodine solution, and the surgical area was isolated. Then, local infiltration anesthesia was established. A full-thickness buccal mucoperiosteal flap was lifted, and the implant socket was drilled. HA was injected into the implant socket by syringe and on the alveolar bone in the experimental group (Figure 1). The implant was inserted at the level of the alveolar ridge (Figure 2), while the other socket of the control group on the opposite side was manipulated normally (Figure 3). Finally, suturing was performed (Figure 4). The principal researcher (WHA) performed all surgical interventions in both groups.

Randomization of the intervention side

Each patient was asked to pick an opaque sealed envelope from a container to allocate the intervention side. The containers included six envelopes with the letter R indicating the right-hand side and six envelopes with the letter L indicating the left-hand side.

Outcome measures

The pain was assessed using the VAS to determine post-implant pain intensity. Each patient was asked to mark their perception of pain at two o’clock in the afternoon on the first, third, and tenth day after implantation. The intensity of pain was classified according to the following categories: 0 = no pain, 1-3 = mild pain, 3-6 = moderate pain, and 6-10 = severe pain [18,19].

Statistical analysis

All statistical analyses were performed using SPSS version 20 (IBM Corp., Armonk, NY, USA). The Kolmogorov-Smirnov test was used to check the normality of the distributions. Levene’s test was used to evaluate the equality of variances. An independent t-test was used to compare the results between the two groups. The significance level was set at 0.05.

## Results

Baseline sample characteristics

**Figure 5:**
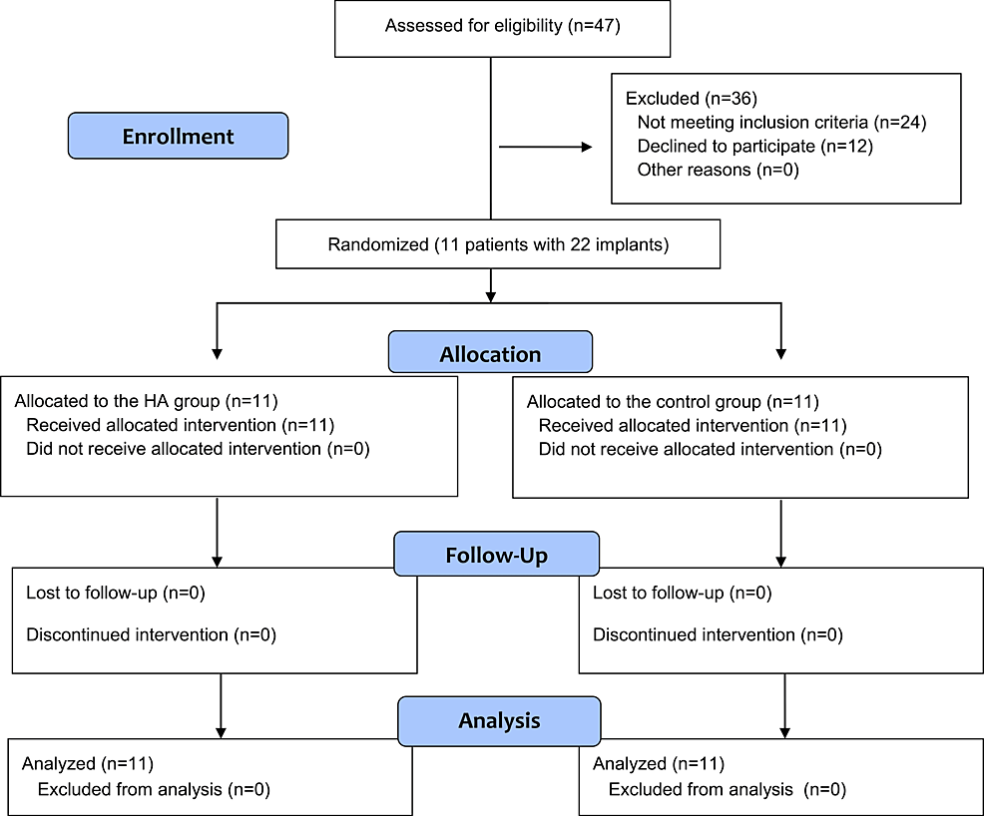
The Consolidated Standards of Reporting Trials (CONSORT) flow diagram of patient recruitment, assignment, follow-up, and inclusion in data analysis.

The Consolidated Standards of Reporting Trials (CONSORT) flow diagram illustrates patient recruitment, assignment, follow-up, and inclusion in data analysis (Figure 5). In total, 11 patients with 22 implants were enrolled (five males (45.45%) and six females (54.54%)) in the study groups, with a mean age of 44 (±11.9) years (Table 1). A total of 11 implant cases were enrolled in the HA group, and 11 implant cases were enrolled in the control group. There were no dropouts.

**Table 1:** Baseline sample characteristics.

Variables	Frequency	Percent
Gender		
Male	5	45.45
Female	6	54.54
Age (years)		
20–39	4	36.36
40–49	4	36.36
50–60	3	27.27
Mean age (in years)	44 ± 11.9	

Perception of pain

In the first assessment time (i.e., on the first day), the greatest VAS score was 6.5 in the HA group and 7.5 in the control group. The lowest VAS score in both study groups was 4. On the third day, the greatest VAS score for both study groups was 3, and the lowest VAS score was 0.5. On the 10th day, the highest VAS score was 0.5 in the HA group and 1 in the control group. The lowest VAS score was 0 in both study groups (Table 2).

**Table 2:** Descriptive statistics of the perceived pain using the visual analog scale (VAS) scores in both groups (n = 11).

Assessment time	Group	Minimum	Maximum	Mean	Standard deviation
First day	Control	4.00	7.50	5.68	1.27
Third day	Control	0.50	3.00	1.72	0.84
Tenth day	Control	0.00	1.00	0.56	0.32
First day	Experimental	4.00	6.50	4.52	1.21
Third day	Experimental	0.50	3.00	1.14	0.96
Tenth day	Experimental	0.00	0.50	0.18	0.25

In the control group, the mean VAS score was 4.52 on postoperative day one. On the third day, it decreased to 1.14, and after 10 days, it further decreased to 0.14. In the control group, the mean VAS score was 5.68 on postoperative day one. On the third day, it decreased to 1.72, and on the 10th day, it further decreased to 0.56. It was observed that at all assessment times, the mean VAS scores in the control group were significantly greater than those in the HA group (p < 0.05) (Table 3).

**Table 3:** The results of applying the t-test for equality of means analysis. Levene’s test was used to evaluate the equality of variances. df: degrees of freedom; SE: standard error of the difference; CI: confidence interval

Assessment times for the comparisons between the two groups		Levene’s test		T-test for equality of means						
		F	P-value	t-value	df	P-value (two-tailed)	Mean difference	SE	95% CI of the difference	
									Lower	Upper
First day	Equal variances assumed	0.001	0.919	0.085	2.0	0.021	1.15	0.52	-0.65	1.56
	Equal variances are not assumed			0.085	1.995	0.025	1.15	0.53	-0.65	1.56
Third day	Equal variances assumed	0.017	0.677	0.047	2.0	0.043	0.18	0.58	-0.62	0.99
	Equal variances are not assumed			0.047	1.969	0.043	0.18	0.58	-0.62	0.99
Tenth day	Equal variances assumed	0.276	0.112	0.129	2.0	0.021	0.18	0.38	-0.11	0.48
	Equal variances are not assumed			0.129	1.704	0.024	0.18	0.38	-0.12	0.48

## Discussion

The study results showed that HA had a positive effect in relieving pain after dental implants, with statistically significant differences between the experimental and control groups at the three assessment times. This may be due to the physiological and structural properties of HA, as it has an anti-inflammatory effect in the initial stages, an antibacterial effect, and activates cell migration, proliferation, and differentiation. As a result, it helps the differentiation of the cells responsible for rebuilding the damaged tissue and accelerates the healing of the bony space at the site of the wound; by stimulating angiogenesis, it also accelerates bone repair.

We found that the application of HA gel can reduce pain in the first and third days after the dental implant, which was also reported by Yıldırım et al. [20]. Their study was conducted on 36 patients requiring a free gingival graft from the palate. The patients were divided into two study groups with the application of HA gel at two different concentrations of 0.2% and 0.8%, followed by covering it with a gingival pad, with nothing placed in the third control group. The pain was recorded using the VAS on days three, seven, 14, 21, and 42, and the results indicated lower pain values in test groups on days three and seven (p < 0.001 and p < 0.001, respectively).

Our results also agreed with the study by Abdelmabood and Eid, who studied the effect of HA gel in relieving pain after applying it to patients with odontogenic cysts [21]. The results showed statistically significant differences between the study groups, and HA reduced pain levels on the first, third, and seventh days after surgery.

Our study findings agreed with those of Yilmaz and colleagues, who used HA gel after extracting third molars [13]. Their results showed a significant decrease in pain in the HA groups according to the VAS (p = 0.001).

Our study differed from the study reported by Marin et al. which included 30 patients with uncontrolled type 2 diabetes who had symmetrical extractions in the mandible [22]. HA gel at a concentration of 0.8% was applied to the alveolar socket of the test group. Pain intensity was recorded on the fifth, 10th, 15th, 20th, and 25th days, and no statistically significant differences were noted. This difference can be explained by applying HA in patients with uncontrolled diabetes prone to infections and delayed wound healing.

Study limitations

There were some limitations of this study. The sample size did not allow for gender-based comparisons. In addition, discrimination between patient responses on the pain scale based on their age group was not done in this study. Therefore, future research work should consider gender- and possible age-related effects on the perception of pain following implantation. This trial evaluated the effect of HA on pain control; however, the effect on the quantity and quality of the newly formed bone around the inserted implants was not assessed. Furthermore, other materials should be compared with HA regarding pain control following dental implantation in future clinical trials.

## Conclusions

This study showed that applying HA in the implant cavity and on the surrounding bone effectively reduced pain after dental implant surgery in comparison with the control group. Patients had lower mean pain scores at one, three, and ten days following surgery compared to the conventional method. This material is suggested to be an adjunctive method to control postsurgical pain after dental implantation. More studies are required to assess the effect of this material on the quality of bone being formed in the post-healing period. Additionally, gender and age differences in pain perception should be evaluated in future research.
